# Supplementary material for: Radiogenomics Analysis Linking Multiparametric MRI and Transcriptomics in Prostate Cancer
Source: Cancers (Basel). 2023 Jun 6;15(12):3074. doi: 10.3390/cancers15123074 (PMC10296397; doi:10.3390/cancers15123074)
Supplement: Supplementary file 1 [file cancers-15-03074-s001.zip › cancers-2382163-supplementary.pdf]

Supplementary material

Table S1. *Overview of MRI acquisition settings in the training dataset.*

	T2W			DWI			DCE		
	**AMC**	**NKI**	**RUMC**	**AMC**	**NKI**	**RUMC**	**AMC**	**NKI**	**RUMC**
**TR (ms)**	4500-6930	5321-10233	4000-6050	4000-4500	3429-4498	2500-4200	50	4.00-5.50	3.85-36.00
**TE (ms)**	108	120	99-104	87	67-69	60-81	4	1.79-2.02	1.40-1.42
**Thickness (cm)**	3	3	3	4	3	3.00-3.60	4	6	3.00-4.50
**Width (voxels)**	512	512	320-512	136	176	84-160	144	176-256	128-160
**Height (voxels)**	512	512	320-512	160	176	128-168	192	176-256	128-160
**Field Strength (Tesla)**	1.50	3.00	3.00	1.50	3.00	3.00	1.50	3.00	3.00
**FA (degrees)**	150	90	117-160	90	90	90	70	8-15	12-14
**Endorectal coil (Yes/No)**	Yes	Yes	No	Yes	Yes	No	Yes	Yes	No
**MRI scanner**	SIEMENS Avanto	Philips Achieva	SIEMENS Skyra/ TrioTim	SIEMENS Avanto	Philips Achieva	SIEMENS Skyra/ TrioTim	SIEMENS Avanto	Philips Achieva	SIEMENS Skyra/ TrioTim
**Pixel Spacing (mm)**	0.31	0.27	0.31-0.62	1.62	1.03	1.40-2.00	1.67	1.02-2.05	1.50-1.62
**Temporal Resolution (s)**							3.11-3.12	3.07-3.67	3.31-4.24
**Contrast agent**							Gadobutrol (0.1mmol/kg)	Gadoterate meglumine (0.1mmol/kg)	Gadobutrol (0.1mmol/kg)

Table S2. *Overview of MRI acquisition settings in the test dataset.*

	T2	DWI	DCE
**TR (ms)**	3141-10233	2856-5355	4.00-5.50
**TE (ms)**	110-120	54-104	1.90-2.02
**Thickness (cm)**	3.00	2.73-3.00	6.00
**Width (voxels)**	256-720	176-336	192-384
**Height (voxels)**	256-720	176-336	192-384
**Field Strength (Tesla)**	1.50-3.00*	1.50-3.00*	1.50-3.00*
**FA (degrees)**	90	90	13-30
**Endorectal coil (Yes/No)**	Yes	Yes	Yes
**MRI scanner**	Philips Achieva	Philips Achieva	Philips Achieva
**Pixel Spacing (mm)**	0.26-0.70	0.56-1.33	1.02-1.36
**Temporal Resolution (s)**			2.42-4.49
**Contrast agent**			Gadoterate meglumine (0.1mmol/kg)

*only one patient was scanned at 1.5T

Table S3 *– Complete list of transcriptomic features consisting of 132 functional features describing 14 intracellular signaling pathways and 118 transcription factor activity, as well as 6 gene signature scores.*

**Type of transcriptomic features**	**Acronym**	**Full name**
Transcription factors	AR	Androgen receptor
	ARNTL	Aryl hydrocarbon receptor nuclear translocator-like protein 1
	ATF1	Activating transcription factor 1
	ATF2	Activating transcription factor 2
	ATF4	Activating transcription factor 4
	ATF6	Activating transcription factor 6
	BACH1	BTB and CNC homolog 1
	CDX2	Caudal type homeobox 2
	CEBPA	CCAAT/enhancer-binding protein alpha
	CEBPB	CCAAT/enhancer-binding protein beta
	CEBPD	CCAAT/enhancer-binding protein delta
	CREB1	CAMP responsive element binding protein 1
	CTCF	CCCTC-binding factor
	E2F1	E2F transcription factor 1
	E2F2	E2F transcription factor 2
	E2F3	E2F transcription factor 3
	E2F4	E2F transcription factor 4
	EGR1	Early growth response 1
	ELK1	ELK1 ETS transcription factor ELK1
	EPAS1	Endothelial PAS domain-containing protein 1
	ERG	Erythroblast transformation specific (ETS) transcription factor ERG
	ESR1	Estrogen receptor alpha
	ESR2	Estrogen receptor beta
	ETS1	Erythroblast Transformation Specific 1
	ETS2	Erythroblast Transformation Specific 2
	ETV4	ETS Variant Transcription Factor 4
	FLI1	Friend leukemia integration 1 transcription factor
	FOS	Fos Proto-Oncogene, AP-1 Trancription Factor Subunit
	FOSL1	FOS like 1, AP-1 transcription factor subunit
	FOSL2	FOS like 1, AP-2 transcription factor subunit
	FOXA1	Forkhead box protein A1
	FOXA2	Forkhead box protein A2
	FOXL2	Forkhead box protein L2
	FOXM1	Forkhead box protein M1
	FOXO1	Forkhead box protein O1
	FOXO3	Forkhead box protein O3
	FOXO4	Forkhead box protein O4
	FOXP1	Forkhead box protein P1
	GATA1	GATA-binding factor 1
	GATA2	GATA-binding factor 2

	GATA3	GATA-binding factor 3
	GLI2	GLI family zinc finger 2
	HIF1A	Hypoxia inducible factor 1 subunit alpha
	HNF1A	Hepatocyte nuclear factor 1 alpha
	HNF4A	Hepatocyte nuclear factor 4 alpha
	HSF1	Heat shock transcription factor 1
	IRF1	IRF1 – interferon regulatory factor 1
	IRF9	IRF9 – interferon regulatory factor 9
	JUN	Jun Proto-Oncogene, AP-1 Transcription Factor Subunit
	JUND	JunD proto-oncogene, AP-1 transcription factor subunit
	KLF4	Kruppel-like factor 4
	KMT2A	Histone-lysine N-methyltransferase 2A
	LEF1	Lymphoid enhancer-binding factor 1
	MITF	Microphthalmia-associated transcription factor
	MYB	MYB Proto-Oncogene, Transcription Factor
	MYC	MYC proto-oncogene, bHLH transcription factor
	MYCN	MYCN proto-oncogene, bHLH transcription factor
	NFATC2	Nuclear factor of activated T cells 2
	NFE2L2	NFE2 like bZIP transcription factor 2
	NFIC	Nuclear factor I C
	NFKB1	Nuclear factor kappa B subunit 1
	NFKB2	Nuclear factor kappa B subunit 2
	NFYA	Nuclear transcription factor Y subunit alpha
	NR2F2	Nuclear receptor subfamily 2 group F member 2
	NR3C1	Nuclear receptor subfamily 3 group C member 1
	NR5A1	Nuclear receptor subfamily 5 group A member 1
	PAX6	Paired box 6
	PAX8	Paired box 8
	PGR	Progesterone receptor
	POU2F1	POU class 2 homeobox 1
	POU2F2	POU class 2 homeobox 2
	PPARA	Peroxisome proliferator activated receptor alpha
	PPARG	Peroxisome proliferator activated receptor gamma
	PRDM14	PR/SET domain 14
	RARA	Retinoic acid receptor alpha
	REL	REL proto-oncogene, NF-kB subunit
	RELA	RELA proto-oncogene, NF-kB subunit
	RELB	RELB proto-oncogene, NF-kB subunit
	REST	RE1 silencing transcription factor
	RFX5	Regulatory factor X5
	RUNX1	RUNX family transcription factor 1
	RUNX3	RUNX family transcription factor 3
	RXRA	Retinoid X receptor alpha
	SMAD3	Mothers against decapentaplegic homolog 3
	SMAD4	Mothers against decapentaplegic homolog 4
	SOX10	SRY-box transcription factor 10

	SOX2	SRY-box transcription factor 2
	SOX9	SRY-box transcription factor 9
	SP1	Specificity protein 1 transcription factor
	SP3	Specificity protein 3 transcription factor
	SPI1	Hematopoietic Transcription Factor PU.1
	SREBF1	Sterol regulatory element binding transcription factor 1
	SREBF2	Sterol regulatory element binding transcription factor 2
	SRF	Serum response factor
	STAT1	Signal transducer and activator of transcription 1
	STAT2	Signal transducer and activator of transcription 2
	STAT3	Signal transducer and activator of transcription 3
	STAT4	Signal transducer and activator of transcription 4
	STAT5A	Signal transducer and activator of transcription 5A
	STAT5B	Signal transducer and activator of transcription 5B
	STAT6	Signal transducer and activator of transcription 6
	TAL1	TAL bHLH transcription factor 1
	TCF3	Transcription factor 3
	TCF7L2	Transcription factor 7 like 2
	TFAP2A	Transcription factor AP-2 alpha
	TFAP2C	Transcription factor AP-2 gamma
	TFDP1	Transcription factor Dp-1
	TP53	Tumor protein p53
	TP63	Tumor protein p63
	TWIST1	Twist family bHLH transcription factor 1
	USF1	Upstream transcription factor 1
	USF2	Upstream transcription factor 2
	VDR	Vitamin D receptor
	WT1	Wilms tumor 1
	YY1	Yin Yang 1
	ZBTB33	Zinc finger and BTB domain containing 33
	ZEB1	Zinc finger E-box binding homeobox 1
	ZNF263	Zinc finger 263
**Pathways***	EGFR	Epidermal growth factor receptor -regulates growth, survival, migration, apoptosis, proliferation, and differentiation in mammalian cells.
	PI3K	Phosphatidylinositol 3-kinase -promotes growth and proliferation.
	Androgen	Involved in the growth and development of the male reproductive organs.
	EGFR	Epidermal growth factor receptor - regulates growth, survival, migration, apoptosis, proliferation, and differentiation in mammalian cells.
	Estrogen	Promotes the growth and development of the female reproductive organs.
	Hypoxia	Promotes angiogenesis and metabolic reprogramming when O_2 levels are low.
	JAK-STAT	Janus kinases (JAKs), signal transducer and activator of transcription proteins (STATs) - involved in immunity, cell division, cell death, and tumor formation.

	MAPK	Mitogen-activated protein kinases (MAPKs) - integrates external signals and promotes cell growth and proliferation.
	NFkB	Nuclear factor kappa B - regulates immune response, cytokine production and cell survival.
	P53	Regulates cell cycle, apoptosis, DNA repair and tumor suppression.
	TGFb	Transforming growth factor beta - involved in the development, homeostasis, and repair of most tissues.
	TNFa	Tumor necrosis factor (TNF) a - mediates hematopoiesis, immune surveillance, tumor regression and protection from infection.
	Trail	TNF-related apoptosis-inducing ligand - induces apoptosis.
	VEGF	Vascular endothelial growth factor - mediates angiogenesis, vascular permeability, and cell migration.
	WNT	Created from the names Wingless and Int-1 - regulates organ morphogenesis during development and tissue repair.
**Signatures**	Decipher geometric mean	Decipher signature, derived using the geometric mean.
	Decipher PCA	Decipher signature, derived using PCA.
	Decipher ssGSEA	Decipher signature, derived using single-sample gene set enrichment analysis.
	PORTOS geometric mean	PORTOS signature, derived using the geometric mean.
	PORTOS PCA	PORTOS signature, derived using PCA.
	PORTOS ssGSEA	PORTOS signature, derived using single-sample gene set enrichment analysis.

*The description here provided for each of the pathways was derived from the PROGENy pathway signatures GitHub repository - https://saezlab.github.io/progeny/articles/progeny.html.
